# Programmable Collective Behavior in Dynamically Self‐Assembled Mobile Microrobotic Swarms

**DOI:** 10.1002/advs.201801837

**Published:** 2019-01-23

**Authors:** Berk Yigit, Yunus Alapan, Metin Sitti

**Affiliations:** ^1^ Physical Intelligence Department Max Planck Institute for Intelligent Systems 70569 Stuttgart Germany; ^2^ School of Medicine and School of Engineering Koc University 34450 Istanbul Turkey

**Keywords:** collective systems, dynamic self‐assembly, microrobotics, reconfigurable systems, swarm control

## Abstract

Collective control of mobile microrobotic swarms is indispensable for their potential high‐impact applications in targeted drug delivery, medical diagnostics, parallel micromanipulation, and environmental sensing and remediation. Without integrated electronics for sensing and actuation, current microrobotic systems should rely on physical interactions among individual microrobots for local communication and cooperation. Here, it is shown that mobile microrobotic swarms with well‐defined collective behavior can be designed by engineering magnetic interactions among individual units. Microrobots, dynamically self‐assembled from magnetic microparticles into linear chains, locomote on surfaces in response to a precessing magnetic field. Control over precessing magnetic field allows engineering attractive and repulsive interactions among microrobots and, thus, collective order with well‐defined spatial organization and stable parallel operation over macroscale distances (≈1 cm) and through confining obstacles. The design approach described here addresses programmable assembly, propulsion, and collective behavior of dense mobile microrobot swarms simultaneously by engineering magnetic interactions and dynamic actuation of microrobots. The presented approach will advance swarm microrobotics by enabling facile and rapid formation of self‐organized and reconfigurable microrobotic swarms with programmable collective order and stability.

## 1 Introduction

Operation of functional microrobots as individual units, as well as in swarms, has the potential to revolutionize manipulation of the microscopic world. Various microrobots, actuated via magnetic, electrical, and biological means, have been developed and proved significant potential in cargo transportation, micromanipulation, microsurgery, sensing, environmental remediation, and drug delivery applications at the microscale (<100 µm).^1^, ^2^, ^3^, ^4^, ^5^, ^6^, ^7^, ^8^ However, considering the size difference among microrobots and their targets, such as tissues and organs, capacity of an individual microrobot would be insufficient for achieving desired effects at the macroscale (≈1 cm). Therefore, many real‐world applications would require microrobots to operate in parallel to amplify functional throughput, without hindering the motility and function of one another.^9^ Furthermore, microrobotic collectives would be able to cooperate in large groups to generate higher order functionalities that is required to accomplish complex tasks, going well beyond the capability of an individual microrobotic unit.^9^ In order to achieve this goal, physical communication among microrobots through local physical interactions are required in the absence of onboard computational and sensing capabilities.^10^, ^11^, ^12^, ^13^ Therefore, programming collective interactions at the microscale requires fundamental understanding and engineering of physical interactions among microrobots, which remains to be a significant scientific challenge.

Swarming synthetic and biohybrid microrobots have been investigated for micromanipulation and medical applications.^7^, ^14^, ^15^, ^16^, ^17^, ^18^, ^19^, ^20^, ^21^ In general, the main strategy for controlling swarms relies on the motile response of microrobotic units to remotely controlled global fields, such as magnetic fields. Magnetotactic bacteria swarms were used to manipulate microobjects to assemble larger structures, although swarm dynamics was governed by global magnetic field gradients instead of interactions among bacteria.^16^ Similarly, synthetic helical microswimmer aggregates, actuated with global magnetic fields, were shown to enhance real‐time signals for medical imaging modalities.^7^, ^17^ Recently, swarms of reconfigurable microrobots formed using magnetic micro/nanoparticles were used as lab‐on‐a‐chip biosensing systems,^22^, ^23^ hyperthermia agents,^19^ and for mechanical lysis of fibrin gels.^18^ Despite all these advances in fabrication of microrobot swarms, controlling dense mobile microrobot swarms with internal collective order remains a big challenge in the field,^9^, ^24^ because typically dense magnetic particle swarms form aggregates under magnetic fields. Therefore, physical interactions among microrobots are still yet to be elucidated for engineering ordered collective motion, including spatial organization of microrobots and their parallel operation.

Recent advances in bottom‐up magnetic colloidal assembly have enabled engineering microrobots with controlled size, shape, and function.^25^, ^26^, ^27^, ^28^ However, collective operation of such magnetically self‐assembled microrobots in large numbers suffer from uncontrolled aggregation of robots due to magnetic attractions among individuals. Here, we show that microrobotic swarms with well‐defined collective order can be fabricated via dynamic self‐assembly of magnetic particles into mobile microrobotic linear chains that communicate with each other through engineered magnetic interactions. Such self‐assembled microrobots are generated simultaneously in large numbers and their propulsion near a solid surface can be driven and controlled using precessing magnetic fields. Microrobot swarms can be reversibly assembled and disassembled by applying and removing the external magnetic field on‐demand. Once assembled, precise control over magnetic field allows engineering the collective behavior of microrobots through tuned pairwise magnetic dipole–dipole interactions. We show that these microrobotic swarms can be guided through confined environments, without compromising their structural and functional integrity. Furthermore, these motile microrobotic arrays can be used to generate directional flows and transport cargoes of various sizes (1–20 µm) on a surface and inside a bulk fluid. Design approach described here addresses programmable assembly, propulsion, and collective behavior of dense mobile microrobot swarms simultaneously by engineering magnetic interactions and dynamic actuation of microrobots. This system demonstrates a blueprint toward the next generation of rapid, reconfigurable, and reversible assembly of functional microrobotic swarms with well‐defined collective order.

## 2 Results

### 2.1 Dynamic Self‐Assembly of Mobile Microrobots and Their Pairwise Interactions

**Figure 1:**
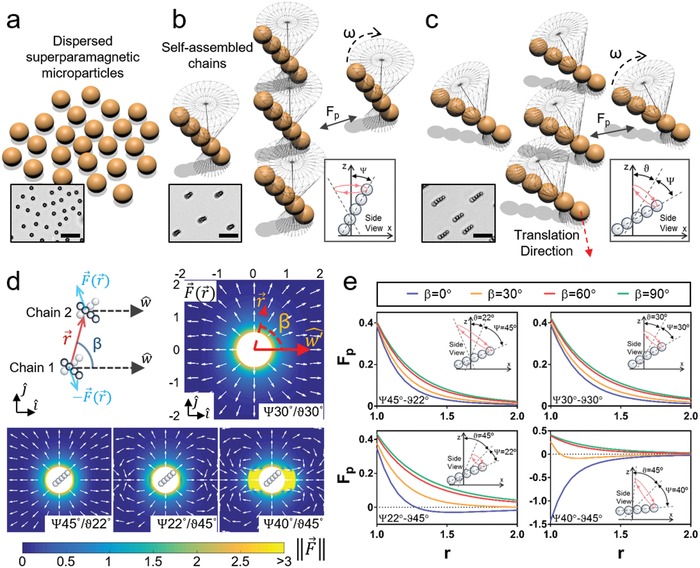
Design of collective microrobotic swarms. a) Magnetic microrobots are composed of superparamagnetic microparticles and b) self‐assemble into chains simultaneously in large numbers via an applied rotating magnetic field with a precession angle (ψ). In‐situ formed chains rotate around their precession axes with a rotational velocity (ω) and apply magnetically repulsive or attractive forces to one another (*F_p_*). c) Self‐assembled chains locomote on a solid surface, with a tilt angle (ϑ) applied to the precession axis in the presence of magnetic inter‐chain forces. Insets at the bottom‐left show typical experimental images illustrated in (a–c). Scale bars are 25 µm. Insets at the bottom‐right demonstrate applied tilt and precession angles from side view. d) Numerical analysis of time‐averaged magnetic dipolar interaction forces between two chains *F*(*r*) depending on inter‐robot positions *r*. Precession axis' projection on the *xy*‐plane ($\hat{w}$) is oriented along the x‐axis. Vectors, indicated by white arrows, show the direction of *F*(*r*) imposed by the first chain that is located at the origin (0, 0) on a virtual second chain positioned at $r\;=\;x\hat{i}+y\hat{j}$. Color bar shows the magnitude of *F*, normalized by characteristic chain interaction force *F*_0_. Chains depicted in the center of each plot represent the approximate area swept by a single chain. e) Quantification of magnetic forces acting along the axis between two microrobotic chains $\left(F_{P}=\;F\;\times \;\hat{r}\right)$ depending on their distance (*r*), normalized by one chain length, and the relative positioning of chains, indicated by β. Positive *F_p_* values indicate repulsion, whereas negative ones indicate attraction between units. Insets in the plots show prescribed tilt and precession angles for each analysis.

Microrobots were constructed using superparamagnetic microparticles (around 5 µm diameter) and applied magnetic fields, resulting in self‐assembly of microparticles into linear chain structures (**Figure** **1**a,b). Initially, particles are sedimented over a planar solid surface (≈2.5 × 10^3^ particles mm^−2^) whose surface normal is denoted by ***n***. Upon application of a global magnetic field (***B***), induced magnetic dipole moments of superparamagnetic microparticles align with the applied field and particles attract each other along their dipoles. Therefore, when a magnetic field precessing about a precession axis ***w*** with a semicone angle (ψ = acos(***B*.*w***) = 70°) is applied at a small angular frequency (ω/2π = 1 Hz), particles assemble into chains (Figure S1, Supporting Information; Movies S1, and S2, Supporting Information). Chain growth results from the dynamic self‐assembly process, which is controlled by magnetic field strength, frequency, precession angle, fluid viscosity, and paramagnetic susceptibility of the particles. As chains grow, hydrodynamic torque due to chain rotation increases, which provides an upper limit for elongation of chains.^25^, ^29^, ^30^ Once assembled, chains revolve about their centers following the conical path of the magnetic field, which can be adjusted by varying ψ (ψ = 0°–90°) (Figure 1b). Upon application of a tilt angle (ϑ = acos(***w*.*n***) > 0°) to the precession axis of the magnetic field, self‐assembled microrobots locomote on the surface (Figure 1c and Movie S2, Supporting Information). Upon removal of the applied magnetic field, chains disassemble into individual beads (Movie S2, Supporting Information).

Time‐dependent forces arise among microrobot chains due to magnetic dipole–dipole interactions. The magnitude and direction of magnetic forces are determined by the precession and tilt angles of the precessing magnetic field, which results in a net attractive or repulsive interaction among chains when averaged over a rotational cycle (Figure S2, Supporting Information). We performed our experiments at various combinations of tilt and precession angles that represent the broad workspace of field configurations 0° ≤ Ψ, ϑ ≤ 90° (Figure S2, Supporting Information), while focusing on the cases where the sum of tilt and precession angles does not exceed 90° to prevent chain fragmentations due to direct collisions among chains and surface (Figure 1d,e). Moreover, selected combinations of tilt and precession angles are around the phase change between repulsive and attractive interactions, which would also provide higher propulsion speeds. Numerical analyses of time‐averaged magnetic dipolar interaction forces ***F***(***r***) between two chains, separated by a relative position vector ***r*** with respect to their centers, are presented in Figure 1d,e. For configurations of ψ = 45°/ϑ = 22° and ψ = 30°/ϑ = 30°, simulation results showed repulsive forces in all directions around the microrobots (β = 0°‐360°, where β is the angle between the precession axis and *r* on the *xy*‐plane) (Figure 1d,e). For configurations of ψ = 22°/ϑ = 45° and ψ = 40°/ϑ = 45°, attractive magnetic forces were produced along the direction of the precession axis (0° ≤ β ≤ 30°) and repulsive forces in orthogonal directions (30° ≤ β ≤ 90°) (Figure 1d,e). Magnetic interaction force decays with distance among microrobots as ≈*r* ^−4^ and its magnitude scales with ≈*B* ^2^ *N* ^2^ *a* ^2^, where *N* is the number of beads in a chain and *a* is the radius of the magnetic particles (Figure 1e, see Supporting Information for details). Overall interaction direction (attractive/repulsive) is unaffected by the chain length. These results show that physical interactions among individual microrobots can be engineered by tuning precession and tilt angles of applied magnetic fields.

### 2.2 Motility and Steering of Individual Microrobots

**Figure 2:**
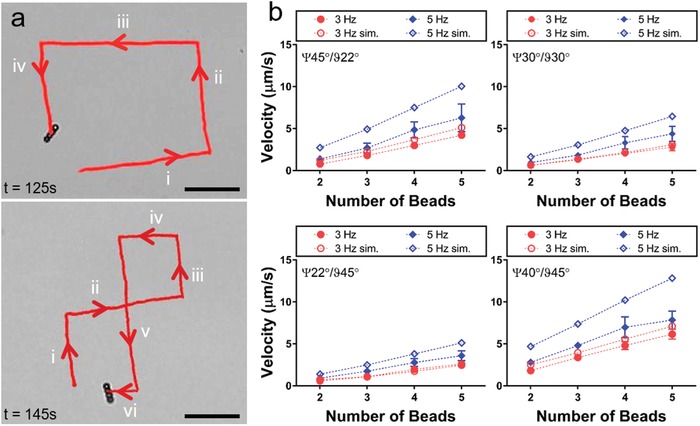
Characterization of single microrobot motility. a) Propulsion of a single microrobot, consisting of self‐assembled microparticles, can be guided by changing orientation of the precession axis. Scale bars are 200 µm. b) Velocity of a single microrobot depends on number of beads in the chain and frequency of the rotating magnetic field, as well as tilt and precession angles. Closed and open symbols correspond to experimental measurements and simulation results, respectively. Error bars represent the standard deviation.

Upon application of a tilt angle to the precession axis, rotating microrobotic chains can locomote on the surface and direction of locomotion can be controlled by changing orientation of the precession axis along the *xy*‐plane (**Figure** **2**a and Movie S3, Supporting Information). Velocity of individual chains increases with the length of the chain, actuation frequency, and applied tilt and precession angles (Figure 2b). Experimental results indicated that change in the precession angle had a greater influence on the chain velocity compared to the tilt angle. These results show that locomotion of individual microrobotic chains can be guided and their velocities can be tuned by changing the chain length and frequency.

Propulsion of microrobotic chains is mediated by a mismatch of hydrodynamic mobility between two ends of the chain. This is due to the presence of a hydrodynamic no‐slip boundary, which alters the mobility of immersed particles as a function of their distance from the surface.^31^, ^32^ Contact friction and adhesion between particles and glass substrate are assumed to be negligible due to a thin surfactant layer and electrostatic repulsion, which lets particles to slide over the surface.^33^ Due to wall induced hydrodynamic symmetry‐breaking, in‐plane component of magnetic torque on a chain is translated into linear motion^25^, ^34^ (see Supporting Information for a mechanistic explanation of propulsion; Figure S3, Supporting Information). Indeed, numerical modeling of the chain dynamics, incorporating effects of magnetic interactions, hydrodynamics including wall effects, gravity, and solid body collisions, showed that chain translation is plausible based on the proposed mechanism (Movie S4, Supporting Information). Translation velocity increases almost linearly with angular frequency and number of beads in a chain, which matches with the experimental observations. Based on our measurements, our system operates in a regime where the amplitude and frequency of chain precession follows that of the magnetic field closely (up to 10 Hz for B<10 mT, Figure S4a–c, Supporting Information). Beyond a critical frequency, sweep radius of chains decrease and velocity of chains is no longer proportional to the field frequency (Figure S4d, Supporting Information). Furthermore, simulated chain velocities were in agreement with the experimental observations for all tilt and precession angle combinations for different frequencies (Figure 2b).

For applications involving navigation of swarms, propulsion direction of chains needs to be controlled. Translation direction of microrobots can be controlled by changing the precession axis' orientation along the *xy*‐plane. Chain translation direction is in the *xy*‐plane and it is orthogonal to the precession axis with a small offset (Figure S5, Supporting Information and Movie S3, Supporting Information).

### 2.3 Collective Order in Microrobotic Swarms

**Figure 3:**
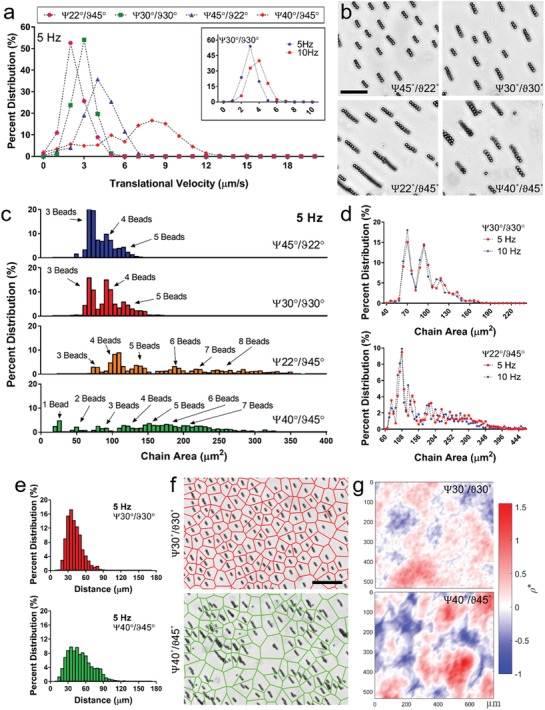
Collective order in self‐assembled mobile microrobotic swarms. a) Velocity histogram of microrobotic swarms at varying tilt and precession angles. Inset shows shift in velocity histogram with increased frequency. b) Typical microscopy images of microrobotic swarms formed at varying tilt and precession angles. Scale bar is 50 µm. c) Percent distributions of chain area in microrobotic swarms formed at different tilt and precession angles. Chain area corresponds to number of beads in a microrobotic unit, which is indicated over histogram peaks. Microrobotic swarms formed at ψ = 45°/ϑ = 22° and ψ = 30°/ϑ = 30° display a narrower distribution of microrobot morphologies compared to swarms formed at ψ = 22°/ϑ = 45° and ψ = 40°/ϑ = 45°. d) Comparison of chain area histograms for microrobotic swarms actuated at 5 and 10 Hz. e) Nearest neighbor distribution, as a measure of inter‐robot separation, showed a more distinct and narrow peak for microrobotic swarms formed at ψ = 30°/ϑ = 30° compared to ψ = 40°/ϑ = 45°, with a broader spectrum. f) Voronoi diagrams and g) normalized density field (ρ*) showed a more uniform spatial distribution for microrobotic swarms formed at ψ = 30°/ϑ = 30° compared to ψ = 40°/ϑ = 45°. Zero corresponds to the mean. Scale bar is 50 µm.

Pairwise interactions among individual microrobots and their motility determine the collective behavior when greater numbers of microrobots are present in a swarm configuration. We characterized velocity of microrobotic swarms constructed using varying precession and tilt angles (**Figure** **3**a and Figure S6, Supporting Information). Mean velocities of microrobot swarms matched with individual chain velocities actuated using same precession and tilt angles. However, the distribution of chain velocities among the population was narrow for configurations of ψ = 45°/ϑ = 22°, ψ = 30°/ϑ = 30°, ψ = 22°/ϑ = 45°, whereas a broad range of velocities was observed for configuration of ψ = 40°/ϑ = 45° (Figure 3a and Figure S6, Supporting Information).

Distribution of microrobot morphologies, measured in number of beads per microrobot, in a swarm population strongly depends on applied tilt and precession angles (Figure 3b,c). A narrower distribution of microrobot morphologies was observed in swarms formed at ψ = 45°/ϑ = 22° and ψ = 30°/ϑ = 30°, with almost all of the chains composed of 3–5 beads. However, swarms formed at ψ = 22°/ϑ = 45° and ψ = 40°/ϑ = 45° displayed a broader distribution of microrobot morphologies, displaying a mixture of short and long chains, as well as thicker aggregates (Figure 3c; Figure S7, Supporting Information and Movie S5, Supporting Information). Reason behind this aggregation is that when two chains come into close proximity under attractive interactions, chains merge. Actuation frequency was observed to have little effect on distribution of chain morphologies (Figure 3d and Figure S7, Supporting Information).

We further characterized spatial distribution of microrobotic swarms using nearest neighbor analysis (Figure 3e and Figure S8, Supporting Information). Swarms formed at ψ = 30°/ϑ = 30° showed a narrower distribution of nearest neighbor distances, indicating an even spatial distribution of microrobots, compared to swarms formed at ψ = 40°/ϑ = 45°. This discrepancy in spatial distribution between ψ = 30°/ϑ = 30° and ψ = 40°/ϑ = 45° was further visualized using Voronoi diagrams (Figure 3f) and density field plots (Figure 3g). Voronoi cell reconstruction (partitioning of area into cells based on nearest neighbor distances) revealed that microrobots are evenly distributed. These results show that microrobots with unidirectional repulsive interactions (i.e., ψ = 45°/ϑ = 22° and ψ = 30°/ϑ = 30°) form swarms with well‐controlled population characteristics including morphology, velocity and spatial organization over large time scales (>30 min) and distances (≈1 cm). On the other hand, presence of directional attractive interaction among chains in a swarm leads to aggregation of smaller chains into larger aggregates with less‐defined morphologies, leading to a larger variance in velocity and spatial distributions (**Table** **1**).

**Table 1:** Key characteristics of collective order for applied precession and tilt angles

Tilt and precession angles	Interaction type	Velocity (Mean ± SD)	Morphology distribution	Spatial distribution
ψ = 45°/ϑ = 22°	Repulsive	Mid (4.2 ± 1.2 µm s^−1^)	Homogeneous	Uniform
ψ = 30°/ϑ = 30°	Repulsive	Mid‐low (3 ± 0.7 µm s^−1^)	Homogeneous	Uniform
ψ = 22°/ϑ = 45°	Attractive	Low (2.3 ± 0.8 µm s^−1^)	Heterogeneous	Nonuniform
ψ = 40°/ϑ = 45°	Attractive	High (6.9 ± 2.9 µm s^−1^)	Heterogeneous	Nonuniform

### 2.4 Locomotion of Microrobotic Swarms through Confined Environments

**Figure 4:**
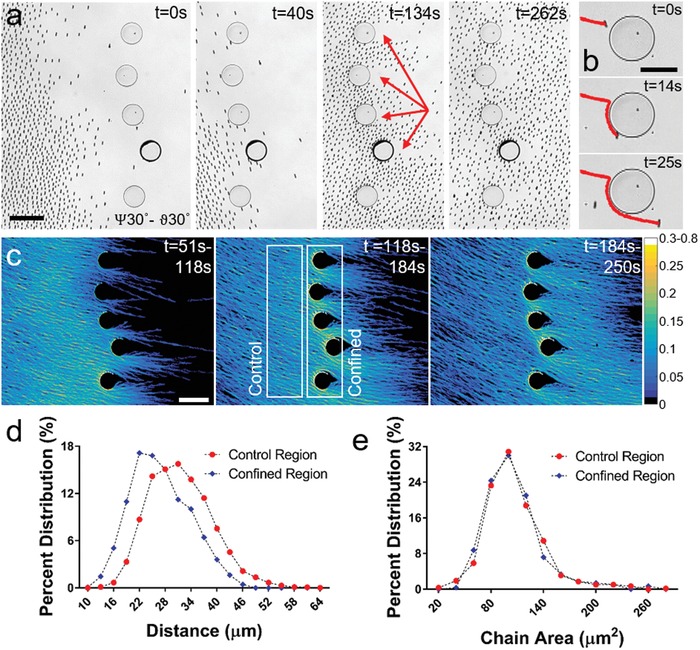
Locomotion of microrobotic swarms through confined environments. a) Microrobotic swarm traversing an array of obstacles displays a compressible behavior, where the swarm is compressed in confinement and expands after obstacles. Microrobots form wakes behind obstacles indicated with red arrows. Scale bar is 200 µm. b) Individual chains, when encountered with the wall boundary, slip over the obstacle in counterclockwise direction and continue in the direction of magnetic actuation when the contact is lost. Scale bar is 100 µm. c) Measurement of microrobot density averaged over discrete time intervals reveals increased swarm density in narrowing confinements. Scale bar is 250 µm. d) Nearest neighbor analysis indicates increased microrobot density in confined region compared to control. e) Comparison of chain area histograms for control and confined regions reveals that chain morphologies are preserved when the swarm is travelling through the obstacles.

Adaptation of microrobotic swarms to their confined local environment, without compromising their integrity and functionality, is a crucial step toward real‐world applications. To test dynamic adaptation of our microrobot swarms to the constraints in their environment, they were guided through an array of convex obstacles forming varying confined spaces in‐between (**Figure** **4**; Figure S9, Supporting Information and Movie S6, Supporting Information). When traversing obstacles, the swarm was compressed and expanded once past the obstacles, demonstrating compressibility of swarms (Figure 4a). Individual microrobots colliding with the obstacle walls were observed to slide on the walls in counterclockwise direction and, once the contact with the wall was lost, continued freely in the direction of magnetic propulsion (Figure 4b). Quantification of microrobot density over discrete time intervals showed increased densities at the confined regions, particularly around the periphery of the obstacles (Figure 4c). An increase in density was also evident in the distribution of nearest neighbor distances in the confined region compared to the control without any geometric confinements (Figure 4d). Furthermore, comparison of chain area histograms for control and confined regions indicated preserved chain morphologies when the swarm was traversing obstacles (Figure 4e). These results show that microrobotic swarms with engineered inter‐robot physical interactions can navigate through simple porous environments (e.g., arrays of convex obstacles), while preserving their structural and functional integrity.

### 2.5 Controlled Cargo Transport by Mobile Microrobotic Swarms

**Figure 5:**
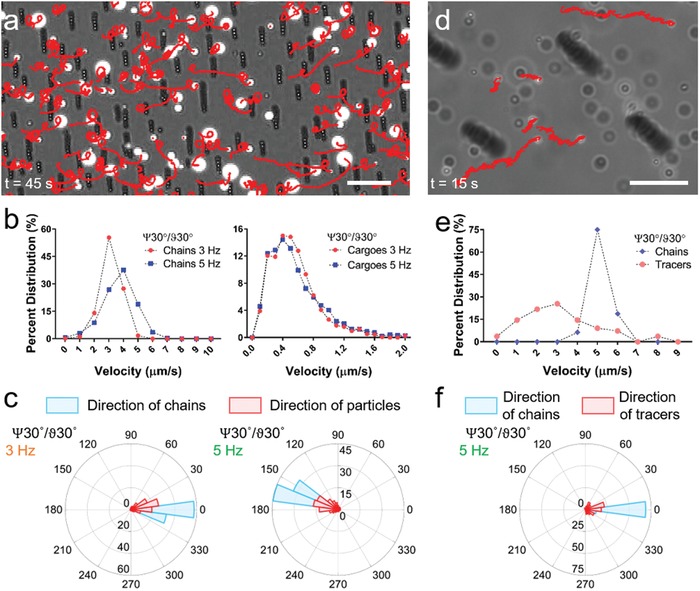
Surface and bulk fluid transport induced by mobile microrobotic swarms. a) Trajectories of large cargoes (5–20 µm particles) transported by the microrobot swarm. Scale bar is 50 µm. b) Velocity histograms for both microrobotic chains and cargoes when chains were actuated at 3 and 5 Hz. c) Polar histograms for microrobotic chains and cargoes demonstrate directed transport of cargoes in the same direction of chain locomotion at 3 and 5 Hz. d) Noncontact transport of small cargoes (1 µm tracer particles) in bulk fluid by flows generated by surface propulsion of microrobotic swarm. Scale bar is 20 µm. e) Velocity histograms for microrobot swarm and tracer particles. f) Polar histogram for microrobot chains and tracer particles shows directed transport of cargoes in bulk fluid.

Compared to individual microrobot units, collective microrobotic swarms can accomplish tasks in parallel that can increase their functional throughput. To demonstrate parallel cargo transportation capability of microrobotic swarms, we introduced large (5–20 µm particles) and small (1 µm tracer particles) cargoes among arrays of microrobots (**Figure** **5** and Movie S7, Supporting Information). Calculated trajectories of cargoes (Figure 5a) showed a transport velocity up to 2 µm s^−1^ (Figure 5b), while microrobots maintained an average translational velocity of 3 and 4 µm s^−1^ when actuated at 3 and 5 Hz, respectively (Figure 5b). In addition, polar distribution histograms for chain and cargo translation direction were overlapping with some orientational spread, indicating directional transport of cargoes near the surface (Figure 5c). Moreover, directionality of the transport was further confirmed by changing swarm translation direction and observing that the cargo transportation direction changed accordingly (Figure 5c). When microrobot swarms were mixed with small 1 µm tracer particles, quantified trajectories (Figure 5d) displayed an average translational velocity of 5 µm s^−1^ for chains actuated at 5 Hz, while the average transport velocity of tracer particles was around 3 µm s^−1^ (Figure 5e). Similar to cargo transport near the surface, transport of tracer particles in the bulk fluid was also directional, as shown by polar distribution histograms for chain and cargo translation directions (Figure 5f). Larger cargoes were sedimented on the substrate, where transportation was due to a mixed effect of collision and convection generated by drag of the mobile chains (Movie S7, Supporting Information). On the other hand, small tracers were suspended in bulk fluid where they were being translated by the hydrodynamic flow generated by the mobile chains (Movie S7, Supporting Information). These results show that when chain microrobots are actuated in high numbers in a swarm configuration, directional transport of large cargoes near the surface and small cargoes in the bulk fluid can be achieved. Such transport phenomena using mobile chain microrobots are reminiscent of cilia found in living systems, which have also inspired fabrication of biomimetic artificial cilia.^35^, ^36^

## 3 Discussions and Conclusion

In nature, communication and cooperation of individual organisms, such as cells, fishes or insects, in large groups facilitate achievement of complex tasks that would be impossible for the individuals. More interestingly, local communication between limited individuals, which do not possess the higher order information of the group, can give rise to complex global behavior that is vital for navigation, preying, foraging, and survival.^37^ Inspired by nature, robotic swarms have been developed at centimeter scales, which allow decentralized simple and modular units to be reconfigured into a team via local interactions achieved by using onboard microcontrollers and infrared sensors.^38^ Despite advances in computational techniques, which could be advantageous at the macroscale, miniaturization of such approaches down to microscale swarms presents significant challenges, due to lack of onboard microcontrollers, powering, and sensors on current mobile microrobots.^10^ Therefore, in design of microrobotic swarms, local communication of individual units need to rely on physical interactions, which is also the case for some biological swarms, such as hydrodynamic interactions among swimming bacteria.^39^ Here, we report an example of such a microrobotic swarm with well‐defined collective order enabled by physical communication of individual robots via engineered magnetic interactions.

The microrobots described here were constructed by dynamic self‐assembly of magnetic particles with applied rotating magnetic fields. Propulsion mechanism of these self‐assembled microrobots relied on symmetry breaking by exploiting the nearby surface. Other microrobots utilizing similar mechanisms have been reported previously, including surface walkers,^25^ microwheels,^18^, ^26^ kayaks,^40^ spinning aggregates,^41^ and nanorod‐sphere propellers.^27^ Although these microrobots were able to show advanced features, including fast propulsion, flow generation, and rolling against gravitational forces, collective operation of such microrobots in high concentrations may not be feasible due to formation of large aggregates, because of unintended magnetic interactions among units. Precise control over magnetic field precession in this study allows engineering magnetic interactions between each unit, which prevents aggregation of microrobots even when compressed as a group in confined spaces.

While previous work has focused on using self‐assembly of magnetic colloidal assemblies and their propulsion using time‐varying magnetic fields,^23^, ^25^, ^26^, ^27^ their interactions and collective behavior in mobile swarms were not addressed so far. Recently, swarming nanoparticle aggregates have been shown to display reconfigurable swarm boundaries, but their internal structure was highly chaotic consisting of perpetually forming and breaking nanoparticle chains.^19^, ^21^, ^42^ In our work, we focus on swarms with ordered internal structure with well‐defined chain‐type microrobotic building blocks, which differs from previous works. The swarm control approach described here can be extended to any microrobotic unit of any particular geometry, interacting with dipolar interactions. While particle‐particle interactions and propulsion can be realized simultaneously by coupling various design principles,^43^ such as applied fields, geometry, and materials, the approach described here takes advantage of using only dynamic magnetic actuation which address simultaneously swarm assembly, propulsion and collective interactions.

Engineered interactions among microrobots described here mainly rely on magnetic repulsion of microrobots, which we showed to be necessary for maintaining the microrobot integrity. Purely repulsive interactions would eventually lead to spreading of robots over long time scales in open spaces. However, such spreading may be especially useful when operating in confined environments to spread and cover large surface areas. Furthermore, to enhance cohesion of swarms, attractive interactions can be further introduced into the design by using additional decoupled external fields, such as acoustic and electrical fields. By fine selection and tuning of pairwise interactions based on different physical effects, attractive and repulsive forces can be balanced at specific distances, which would result in tightly defined spatial ordering of microrobot clusters.^43^, ^44^, ^45^

Microrobotic swarms with a collective order can enable propulsion over large distances while preserving structure and functions of individual units. Propulsion of chains presented in our study is enabled by the presence of a solid surface (for hydrodynamic symmetry breaking). However, application of this approach on arbitrarily curved surfaces would be challenging since each robot would be facing boundaries at different angles while receiving the same global magnetic input. Therefore, they would have different propulsion and/or different inter‐robotic interaction characteristics. Microrobotic swarms engineered using the design approach described here can hold potential for manipulation applications involving semiplanar surfaces (such as linings of vasculature), including active transport and diffusion of drug molecules and remote heating‐based microsurgery. Overall, we describe a design methodology for designing microrobot swarms with collective order by exploiting physical interactions among individual units.

## 4 Experimental Section

*Experimental Setup*: To characterize dynamic self‐assembly and motility of chain arrays, a custom five‐coil magnetic guidance system was placed on an inverted optical microscope (Zeiss Axio Observer A1, Carl Zeiss, Oberkochen, Germany) (Figure S10, Supporting Information). The coil setup was designed to generate 20 mT in *x*‐ and *y*‐directions, as well as 10 mT in *z*‐direction (out of plane).^2^ Individual coils were controlled independently via a current controller (ESCON 70/10, Maxon Motor AG) and current values were determined by the pre‐calibrated field to current ratios. The generated magnetic field was measured to be uniform within 5 mm from the center of the workspace. In addition, magnetic guidance system housed a microfluidic channel (75 µm height × 6 mm width × 10 mm length) composed of laser cut poly(methyl methacrylate) (PMMA) pieces, encompassing an inlet and an outlet, and double sided tape, defining channel shape and height, attached to a cover glass.^46^ To test locomotion of microrobot swarms through confined spaces, obstacles with a diameter of 120 µm diameter were patterned on cover glasses using a commercial two‐photon lithography platform (Nanoscribe, Eggenstein‐Leopoldshafen, Germany).

*Self‐Assembly of Microrobot Swarms*: Superparamagnetic polystyrene microparticles with around 5 µm diameter (Sigma Aldrich, St Louis, MO) were utilized for self‐assembly of chain arrays. The particles were suspended in 0.1% Tween 20 solution (Sigma Aldrich, St Louis, MO) to prevent any nonspecific aggregations and injected into microchannels. Then, an out of plane magnetic field (in *z*‐axis) was applied to uniformly disperse the microparticles followed by an in plane rotating magnetic field resulting in self‐assembled chains on the surface. Then, pre‐determined tilt and precession angles were applied to the rotating magnetic field, producing mobile microrobot chain arrays with specific inter‐robot attractive or repulsive forces. The experiments were performed at an intermediate initial particle density (≈2.5 × 10^3^ particles mm^−2^) that allowed to obtain a satisfactory density of chains after dynamic self‐assembly, such that chains can interact sufficient enough to observe collective behaviors, meanwhile not overcrowding the workspace (Figure S11, Supporting Information).

*Data Analysis*: Acquired images were processed using Fiji^47^ to identify individual chains, positions, and projected surface areas. A tracking software^48^ was used to reconstruct trajectories of individual chains and their velocities. Nearest neighbor, based on Delaunay triangulation, and field density analyses were performed using custom‐written scripts in MATLAB (MathWorks, Inc., Natick, MA). Density field ρ was obtained by averaging binarized pixel intensities over a window with a diameter of two mean nearest neighbor distances, and was normalized as $\rho^{*}=\;\rho /\bar{\rho }-1$.

*Modeling Pairwise Interactions and Chain Propulsion*: For calculation of magnetic interaction forces among microrobot units, two self‐assembled chains, each consisting of *N* paramagnetic beads with radius *a* and magnetic susceptibility χ, were considered. Under an applied field *B*, a magnetic dipole moment ***m*** was induced for each bead. Two chains interacted with each other via magnetic forces ***F***. Pairwise attractive and repulsive interaction forces *F_p_* were quantified by taking the component of ***F*** acting along the line crossing centers of chains. Two chains separated by one chain length distance between their centers interact with a characteristic magnetic force $F_{0}=\frac{\pi }{12\mu_{0}}\;{\left(\frac{a\chi B}{N}\right)}^{2}$, which was used for normalizing ***F*** and *F_p_* in Figure 1d,e (see Supporting Information for calculation of ***F***_0_).

Chain propulsion velocities were simulated by modeling the dynamics of individual superparamagnetic beads under external fields via

(1) $${\dot{r}}_{i}=M_{ij}\;.\left(F_{j}^{M}+F_{j}^{B}+F_{j}^{W}+F_{j}^{G}\right)$$

where beads interact with magnetic dipole forces (***F*** ^***M***^), particle‐particle (***F*** ^***B***^), and particle‐surface (***F*** ^***W***^) excluded volume forces, and gravitational (***F*** ^***G***^) forces. The grand mobility tensor ***M*** couples the velocities of beads (${\dot{r}}_{i}$) to the forces acting on each bead through contributions of self and pair hydrodynamic mobility tensors including hydrodynamic interactions with the wall surface.^32^

## Conflict of Interest

The authors declare no conflict of interest.

## Supporting information

Supplementary

Supplementary

Supplementary

Supplementary

Supplementary

Supplementary

Supplementary

Supplementary
