# Supplementary material for: Programmable Collective Behavior in Dynamically Self‐Assembled Mobile Microrobotic Swarms
Source: Adv Sci (Weinh). 2019 Jan 23;6(6):1801837. doi: 10.1002/advs.201801837 (PMC6425453; doi:10.1002/advs.201801837)
Supplement: Supplementary file 1 — Supplementary [file ADVS-6-1801837-s002.pdf]

Copyright WILEY-VCH Verlag GmbH & Co. KGaA, 69469 Weinheim, Germany, 2019.

ADVANCED SCIENCE
Open Access

Supporting Information

for *Adv. Sci.,* DOI: 10.1002/advs.201801837

Programmable Collective Behavior in Dynamically Self-Assembled Mobile Microrobotic Swarms

*Berk Yigit, Yunus Alapan, and Metin Sitti**

Copyright WILEY-VCH Verlag GmbH & Co. KGaA, 69469 Weinheim, Germany, 2016.

Supporting Information

Programmable collective behavior in dynamically self-assembled mobile microrobotic swarms

Berk Yigit, Yunus Alapan, and Metin Sitti*

Applied magnetic fields:

We have previously defined precession angle ψ as the angle between precession axis ($\hat{\boldsymbol{w}}$) and magnetic field vector (B), and tilt angle θ as the angle between $\hat{\boldsymbol{w}}$ and the z-axis (orthogonal to the wall surface $\hat{\boldsymbol{n}}$) (Fig. S1). Applied magnetic field is varied in time by revolving the magnetic field vector around the precession axis, with angular frequency $\boldsymbol{\omega}$ via the following mathematical operation:

$$\boldsymbol{B}(\boldsymbol{t}) = \boldsymbol{B_0}(\boldsymbol{I} + \sin(\boldsymbol{\omega t})\,[\hat{\boldsymbol{w}}]_\times + (\mathbf{1} - \cos(\boldsymbol{\omega t}))[\hat{\boldsymbol{w}}]_\times{}^2)\boldsymbol{n_0} \quad \text{(S1)}$$

where B_0 is the magnetic field magnitude, t is time, $\boldsymbol{I}$ is the identity matrix, $[\,]_\times$ is the cross-product operator and $\boldsymbol{n}_0$ is the magnetic field direction vector at $t = 0$.

Magnetic interactions among chains:

For calculation of magnetic interaction forces between microrobot units, we considered two self-assembled chains, each consisted of $\boldsymbol{N}$ paramagnetic beads with radius $\boldsymbol{a} = \mathbf{2.5}\,\boldsymbol{\mu m}$ whose positions are denoted by vector $\boldsymbol{r}$. We assumed that the induced magnetic dipole $\boldsymbol{m}$ of each paramagnetic bead is proportional to and in the same direction as the applied magnetic field (B), and is unaffected by magnetic dipoles of neighboring magnetic beads. Thus, the magnetic dipole moment is given by $\boldsymbol{m} = \frac{\boldsymbol{V\chi B}}{\boldsymbol{\mu_0}}$, where V is the bead volume, χ = 1.3 is the volumetric magnetic susceptibility of paramagnetic beads, and $\boldsymbol{\mu_0}$ is the permeability of free

space. Forces acting between two magnetic dipoles denoted by their subscripts i and j is thus given by:

$$\boldsymbol{F}_{ij}^{M} = \frac{3\mu_0}{4\pi r^4}\left(\frac{\boldsymbol{r}_{ij}}{r}(\boldsymbol{m}_i.\boldsymbol{m}_j) + \boldsymbol{m}_i\left(\frac{\boldsymbol{r}_{ij}}{r}.\boldsymbol{m}_j\right) + \boldsymbol{m}_j\left(\frac{\boldsymbol{r}_{ij}}{r}.\boldsymbol{m}_i\right) - 5\frac{\boldsymbol{r}_{ij}}{r^3}(\boldsymbol{r}_{ij}.\boldsymbol{m}_i)(\boldsymbol{r}_{ij}.\boldsymbol{m}_j)\right) \quad \text{(S2)}$$

where $\boldsymbol{r}_{ij} = \boldsymbol{r}_j - \boldsymbol{r}_i$ is the distance vector and $\boldsymbol{r} = \left\|\boldsymbol{r}_{ij}\right\|$ is the distance between dipoles. Magnetic interaction force between chains is then calculated by summing the magnetic dipole forces acting on each bead of a chain.

$$\boldsymbol{F}(\boldsymbol{t}) = \sum_i^N \sum_j^N \boldsymbol{F}(\boldsymbol{t})_{ij}^{M} \,. \quad \text{(S3)}$$

By averaging the interaction forces over a cycle of magnetic field precession, we obtain the mean interaction force between chains:

$$\boldsymbol{F} = \boldsymbol{\omega}\int_0^{1/\omega} \boldsymbol{F}(\boldsymbol{t})\boldsymbol{dt} \,. \quad \text{(S4)}$$

Observing Eqs. S1 and S2, one can see that magnitude of interaction forces between two chains varies as:

$$F \propto N^2\left(\frac{4\pi}{3\mu_0}\frac{(a^3\chi B)^2}{r^4}\right) . \quad \text{(S5)}$$

For a pair of chains separated by a distance of one chain length ($r = 2Na$), a characteristic magnetic interaction magnitude can be obtained as:

$$F_0 = \frac{\pi}{12\mu_0}\left(\frac{a\chi B}{N}\right)^2 . \quad \text{(S6)}$$

Propulsion of self-assembled chains:

For simulating the dynamics of motile self-assembled chains, one needs to consider the dynamics of individual magnetic beads, which can be modeled by the mobility relation:

$$\dot{\boldsymbol{r}}_i = \boldsymbol{M}_{ij}.(\boldsymbol{F}_j^{M} + \boldsymbol{F}_j^{B} + \boldsymbol{F}_j^{W} + \boldsymbol{F}_j^{G}) \quad \text{(S7)}$$

where interactions between particles arise from magnetic dipole-dipole forces ($\boldsymbol{F^M}$), particle-particle ($\boldsymbol{F^B}$) and particle-wall ($\boldsymbol{F^W}$) excluded volume forces, and gravitational ($\boldsymbol{F^G}$) forces. The grand mobility tensor $\boldsymbol{M}$ couples the velocities of beads ($\dot{\boldsymbol{r}}_{\boldsymbol{i}}$) to the known forces acting on each bead through contributions of self and pair hydrodynamic mobility tensors including hydrodynamic interactions with the wall surface[1].

Following the approach presented in[2], particle-particle and particle-wall excluded volume interactions were modeled with modified Lennard-Jones (LJ) interaction forces:

$$\boldsymbol{F^B} = \frac{\boldsymbol{\epsilon}}{\boldsymbol{r-2a}}\left[\left(\frac{\boldsymbol{\sigma}}{\boldsymbol{r-2a}}\right)^{\boldsymbol{12}} - \left(\frac{\boldsymbol{\sigma}}{\boldsymbol{r-2a}}\right)^{\boldsymbol{6}}\right] \tag{S8}$$

$$\boldsymbol{F^W} = \frac{\boldsymbol{\epsilon}}{\boldsymbol{h-a}}\left[\left(\frac{\boldsymbol{\sigma}}{\boldsymbol{h-a}}\right)^{\boldsymbol{12}} - \left(\frac{\boldsymbol{\sigma}}{\boldsymbol{h-a}}\right)^{\boldsymbol{6}}\right] \tag{S9}$$

with $\boldsymbol{\sigma = 0.1a}$, h being the distance of a bead from the wall, and the depth of the potential well $\boldsymbol{\epsilon}$ chosen to be sufficiently small to neglect the attractive term of the LJ interaction[2]. Gravitational force is simply given by $\boldsymbol{F^G} = -\Delta\boldsymbol{\rho V g\hat{z}}$, where $\Delta\boldsymbol{\rho} = \boldsymbol{0.53\ g/cm^3}$ is the density difference between the particle and the surrounding media, g is the gravitational acceleration and $\hat{\boldsymbol{z}}$ is the unit vector along the z-axis.

Equation S7 is integrated in time with an explicit Euler scheme to obtain trajectories of individual beads in a translating chain. For each parameter configuration, the simulation was run with a time step of $2 \times 10^{-6}(\frac{2\pi}{\omega})$ for 10 cycles of magnetic field precession.

Mechanistic explanation of chain propulsion:

A particle moving in a fluid near a solid surface would be subjected to different hydrodynamic drags based on its distance from the surface. Motion of particles that are closer to the surface is hindered, since the hydrodynamic no-slip boundary at the solid surface decreases the particle's hydrodynamic mobility[1-3]. To gain an understanding of chain propulsion, consider a pure torque is imposed on a chain by applying equal and opposite

forces on its two ends as shown in **Fig. S3a**. Due to the difference in hydrodynamic mobilities at two ends, chain section that is closer to the surface will move with a lower velocity than the upper section, even though both ends are subjected to forces with equal magnitudes. Due to rigid body motion, center of the chain would need to displace with a velocity equal to the velocity difference of its two ends.

In order to gain a more analytical understanding of the mechanism of propulsion, we consider a rigid dumbbell rotating and translating above a planar surface (as depicted in **Fig. S3b**). Dumbbell rotates and translates with its center distanced h_0 above a planar solid surface. Due to rigid body motion, velocity of two beads (beads are labeled by subscript i) can be written as

$$\boldsymbol{V}_i = \boldsymbol{V}_o + \boldsymbol{\omega}_0 \times \boldsymbol{r}_i \tag{S10}$$

where $\boldsymbol{V}_i$ is velocity of beads, $\boldsymbol{V}_o$ and $\boldsymbol{\omega}_0$ are the linear and angular velocities of the dumbbell center and $\boldsymbol{r}_i$ is the bead position relative to the dumbbell center. Under a rotating uniform magnetic field without gradients, we consider that the forces on two beads ($\boldsymbol{F}_\mathrm{i}$) are equal in magnitude and opposite in direction, to maintain a net torque for the rotation of the dumbbell. Therefore,

$$\boldsymbol{F}_1 + \boldsymbol{F}_2 = 0 \tag{S11}$$

For achieving a simple yet sufficient representation of hydrodynamics, we consider that hydrodynamic mobilities of beads can be approximated by their self-mobilities, while neglecting pairwise mobility effects. Thus, we get the grand mobility relation between velocities and forces as

$$\begin{bmatrix} \boldsymbol{V}_1 \\ \boldsymbol{V}_2 \end{bmatrix} = \begin{bmatrix} \boldsymbol{M}_{11} & 0 \\ 0 & \boldsymbol{M}_{22} \end{bmatrix} \begin{bmatrix} \boldsymbol{F}_1 \\ \boldsymbol{F}_2 \end{bmatrix} \tag{S12}$$

$\boldsymbol{M}_{ii}$ is the Blake self-mobility tensor for bead i, which is obtained via the image system for a Stokeslet singularity (viscous flow induced by point force) in the vicinity of a planar no-slip boundary[1,3], and it is given by

$$\boldsymbol{M}_{ii} = \begin{bmatrix} M_{ii,x} & 0 \\ 0 & M_{ii,y} \end{bmatrix} \tag{S13}$$

$$M_{ii,x}(h_i) = \frac{1}{6\pi\mu a}\left(1 - \frac{9}{16}\left(\frac{a}{h_i}\right) + \frac{1}{8}\left(\frac{a}{h_i}\right)^3\right) \tag{S14}$$

$$M_{ii,y}(h_i) = \frac{1}{6\pi\mu a}\left(1 - \frac{9}{8}\left(\frac{a}{h_i}\right) + \frac{1}{2}\left(\frac{a}{h_i}\right)^3\right) \tag{S15}$$

where h_i is distance of particle i from the surface. Considering the kinematics of the system in **Fig. S3b**, where the dumbbell rotates about the z-axis, we get $h_1 = h_0 + a.\cos(\omega_0 t)$ and $h_2 = h_0 - a.\cos(\omega_0 t)$.

Then, rearranging Eq. S12 using Eqs. S10, S11, and S13-S15 we get an expression for the translation velocity of the chain center

$$V_{0,x} = \omega_0 a.\cos(\omega_0 t)\left[\frac{1}{M_{11,x}} - \frac{1}{M_{22,x}}\right]\left[\frac{1}{M_{11,x}} + \frac{1}{M_{22,x}}\right]^{-1} \tag{S16}$$

For translation, the first term in brackets should be non-zero, which is rendered possible by a difference in the hydrodynamic mobilities of the two beads when they have different distances from the wall. By integrating Eq. S16, we find that the net displacement of rotating dumbbell parallel to the wall is enabled by the difference in hydrodynamic mobilities of the two beads according to their distance from the wall. This model can be extended to show that an applied torque in the plane of the underlying substrate would also lead to translation in the case of longer chains and different chain rotations (e.g., at different precession and tilt angles).

Chain translation direction:

The pointing direction $\hat{\boldsymbol{p}}$ of each chain is given by $\hat{\boldsymbol{p}} = \hat{\boldsymbol{w}} \times \hat{\boldsymbol{n}}/||\hat{\boldsymbol{w}} \times \hat{\boldsymbol{n}}||$, and it is in the xy-plane and orthogonal to $\hat{\boldsymbol{w}}$. Chain translation direction is determined by the direction of $\hat{\boldsymbol{p}}$, therefore can be controlled by precession axis' orientation along the xy-plane (controlled by an "orientation angle", $\alpha = \arccos(\hat{\boldsymbol{p}}.\hat{\boldsymbol{\imath}})$, where $\hat{\boldsymbol{\imath}}$ is unit vector in x-axis) **(Fig. S5a, Movie S1)**. In the experiments, we found that the actual translation direction ($\hat{\boldsymbol{t}}$) systematically differs from the pointing direction by a slip angle α_S, hence it would be equal to $\alpha + \alpha_S$ **(Fig. S5a)**. With simulations, we found that the slip angle results from interactions between the sedimenting chains and the solid substrate. To further elucidate the mechanism underlying the slip angle, we analyzed the effect of gravity on slip angle using simulations. Indeed, we found that when the gravity is close to zero, microrobot would translate without any slip **(Fig. S5b)**.

References

1. Swan JW, Brady JF. Simulation of hydrodynamically interacting particles near a no-slip boundary. Physics of Fluids **19**, 113306 (2007).
2. Sing CE, Schmid L, Schneider MF, Franke T, Alexander-Katz A. Controlled surface-induced flows from the motion of self-assembled colloidal walkers. Proc Natl Acad Sci U S A **107**, 535-540 (2010).
3. Blake J, Chwang A. Fundamental singularities of viscous flow. Journal of Engineering Mathematics **8**, 23-29 (1974).

Supplementary Figures

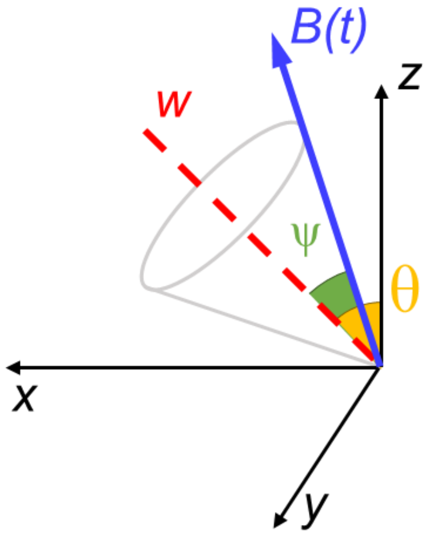

Figure S1. Precessing magnetic field and relevant angles. A time varying magnetic field $\boldsymbol{B}(t)$ precesses in a conical trajectory about a precession axis ($\hat{\boldsymbol{w}}$) with a semi-cone angle ($\Psi = \arccos(\hat{\boldsymbol{w}}.\boldsymbol{B})$). $\hat{\boldsymbol{w}}$ is tilted from the z-axis with a tilt angle ($\theta = \arccos(\hat{\boldsymbol{w}}.\hat{\boldsymbol{n}})$, where $\hat{\boldsymbol{n}}$ is along the z-axis).

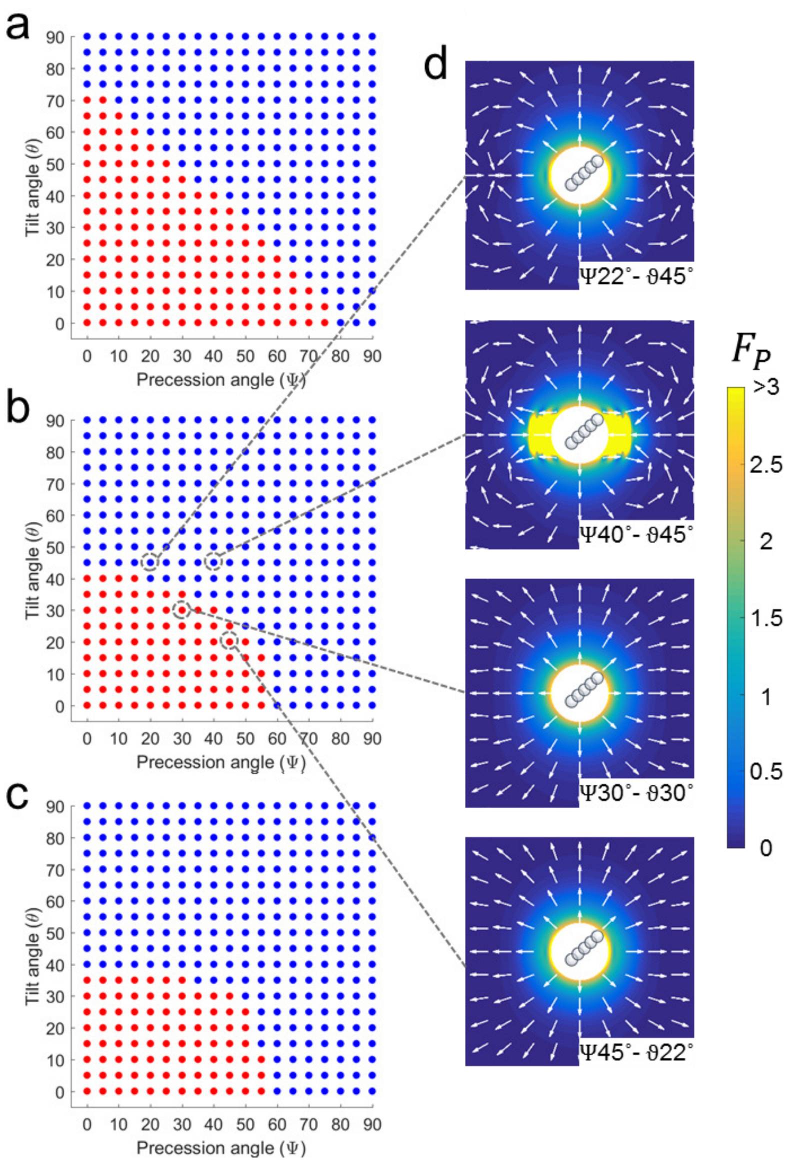

Figure S2. Diagrams for varying tilt and precession angles illustrate two regimes where net pairwise microrobot interactions are attractive (blue) or repulsive (red). Plots represent interactions at **(a)** r=1, **(b)** r=2, **(c)** r=3, where inter-robot distances r are normalized to one chain length. Based on numerical simulations, net interactions are assigned to be attractive if there was at least an angle β with an attractive interaction force (see caption of Fig. 1d for the definition of β), and repulsive if the interaction forces were repulsive at all β. (d) Numerical analysis of time-averaged magnetic dipolar interaction forces between two chains. Color bar shows the normalized magnetic interaction force magnitude, vectors show the direction of the force.

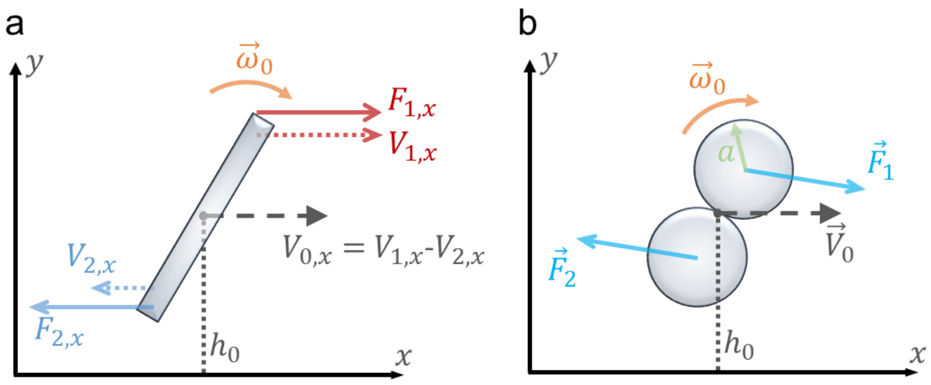

Figure S3. Representation of a simple system explaining the chain propulsion mechanism. (a) Forces with equal magnitudes and opposite directions are applied to the two ends of a chain. The lower section that is closer to the solid surface moves slower than the upper section due to decreased hydrodynamic mobility, which causes the chain to translate to the right. **(b)** Equivalent dumbbell system used for the analytical model, consisting of two beads with radii a.

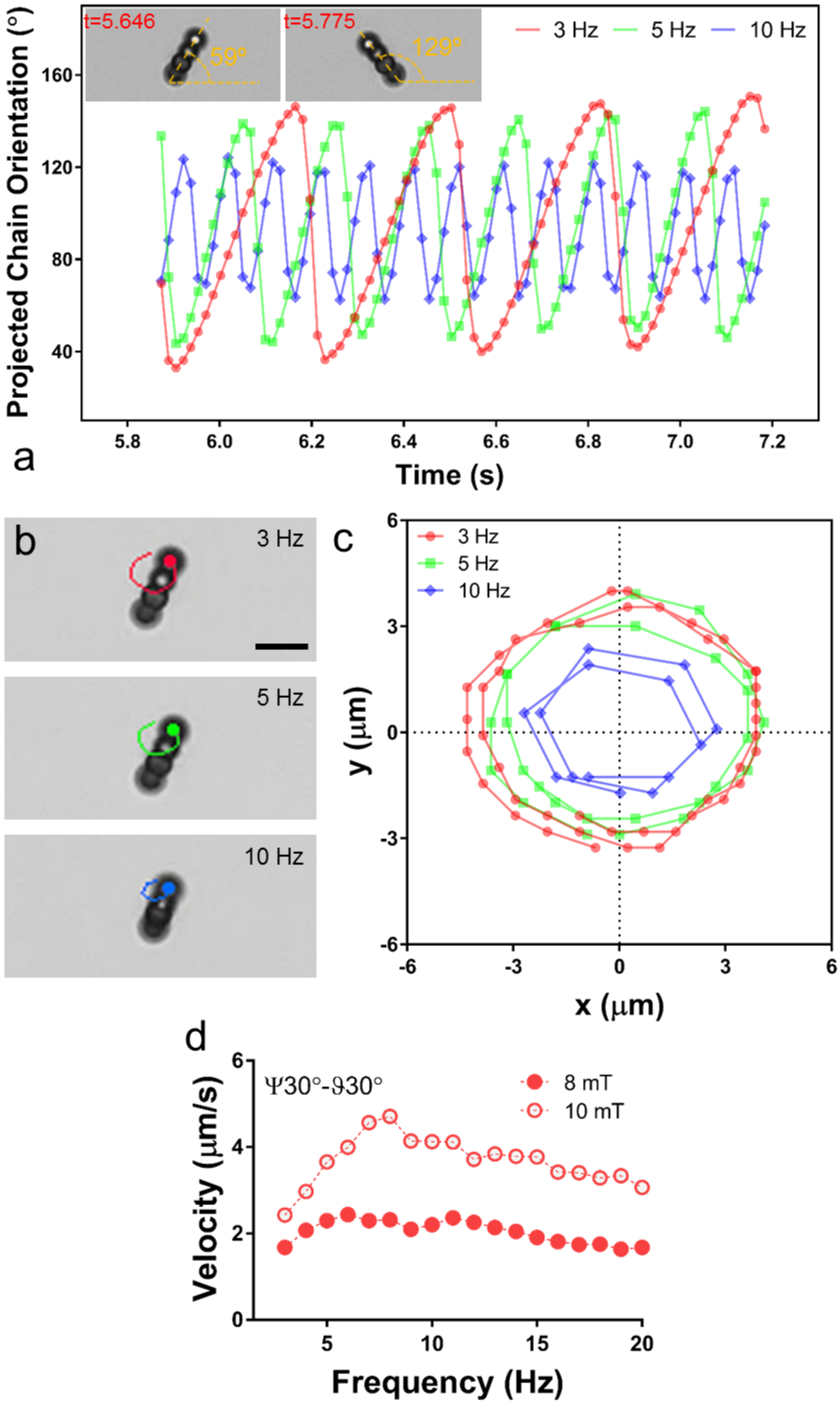

Figure S4. Capability of chains to follow the applied magnetic field at different field frequencies. (a) Rotation of chains exactly follow the frequency of the applied magnetic field measured by the time-dependent change of projected chain orientation. Projected chain orientation angle is shown in the inset. **(b)** Diameter of the conical trajectory that the chains sweep decreases at 10 Hz. Scale bar is 10 μm. **(c)** Tracked positions of upper tip of the chains

at different frequencies shows that sweep amplitude decreases at 10 Hz. **(d)** Experimental characterization of microrobot velocity depending on frequency and magnitude of the applied magnetic field.

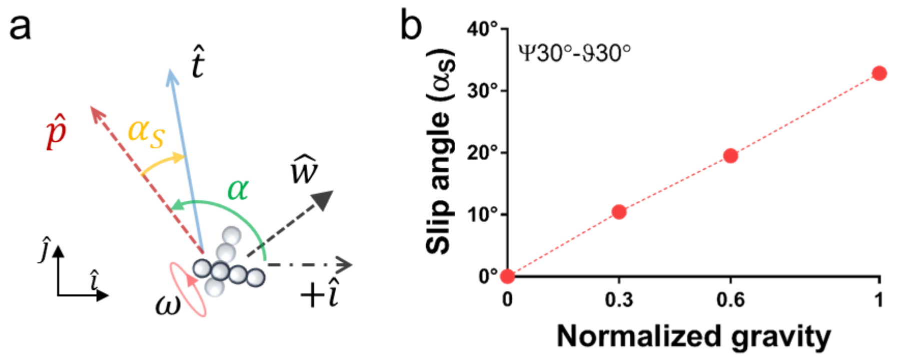

Figure S5. Direction of chain translation. (a) $\hat{\boldsymbol{p}}$: pointing direction, $\hat{\boldsymbol{t}}$: translation direction, $\widehat{\boldsymbol{w}}$: precession axis, $\hat{\boldsymbol{\imath}}$ and $\hat{\boldsymbol{\jmath}}$: unit vectors in x- and y- axes of the lab frame, α: orientation angle, α_S: slip angle. **(b)** Effect of gravity on slip angle based on simulations (normalized gravity, 1= 9.8 m/s^2).

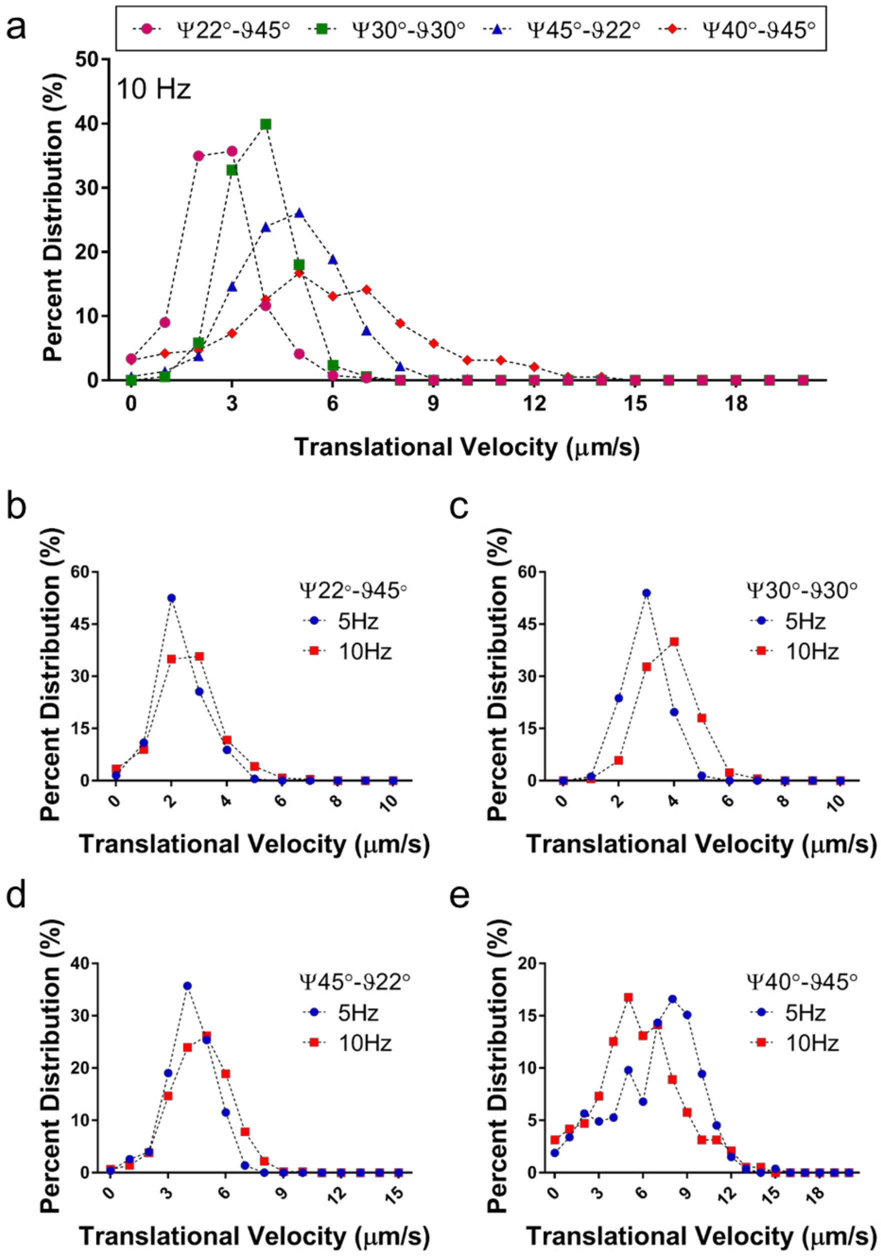

Figure S6. Velocity histogram of microrobot swarms at varying tilt and precession angles and frequencies. (a) Velocity histogram of microrobot swarms at varying tilt and precession angles actuated at 10 Hz. **(b-e)** Velocity histogram of microrobot swarms formed at varying tilt and precession angles actuated at 5 Hz and 10 Hz.

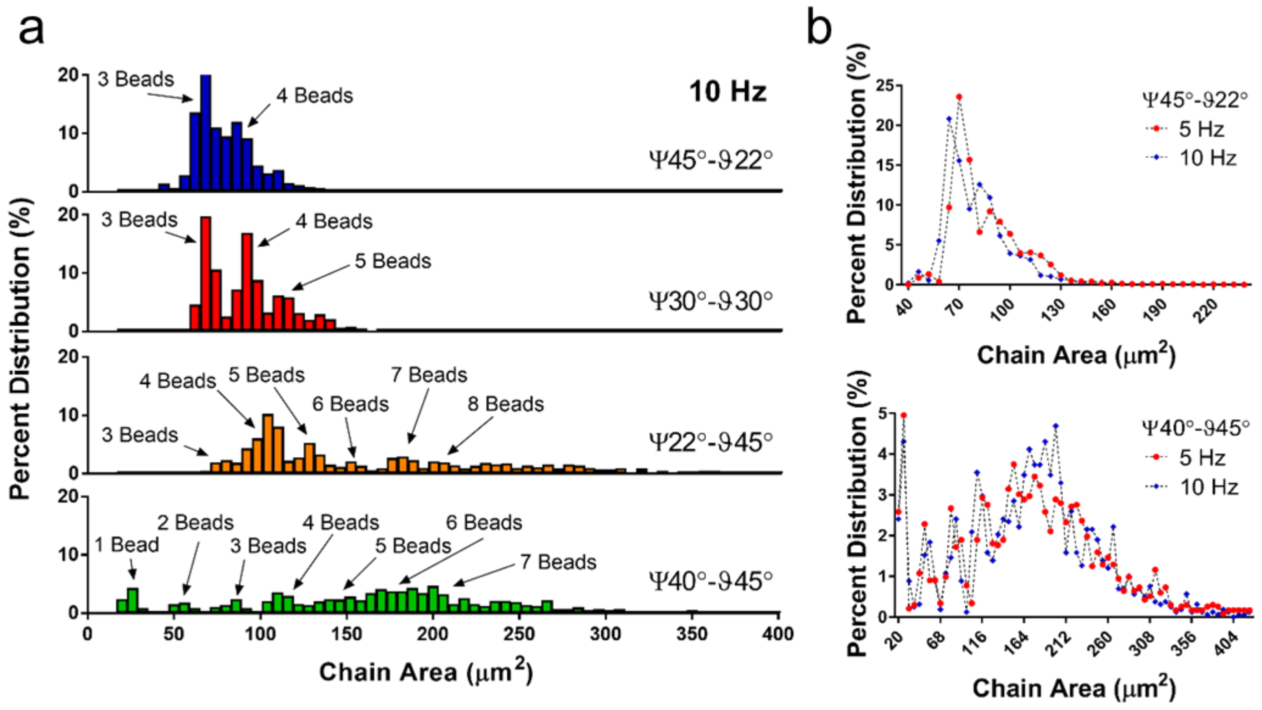

Figure S7. Chain area histograms for microrobot swarms. (a) Percent distributions of chain area in microrobot swarms formed at different tilt and precession angles and actuated at 10 Hz. **(b)** Chain area distributions for microrobot swarms formed at Ψ=45˚-ϑ=22˚ and Ψ=40˚-ϑ=45˚ and actuated at 5 Hz and 10 Hz.

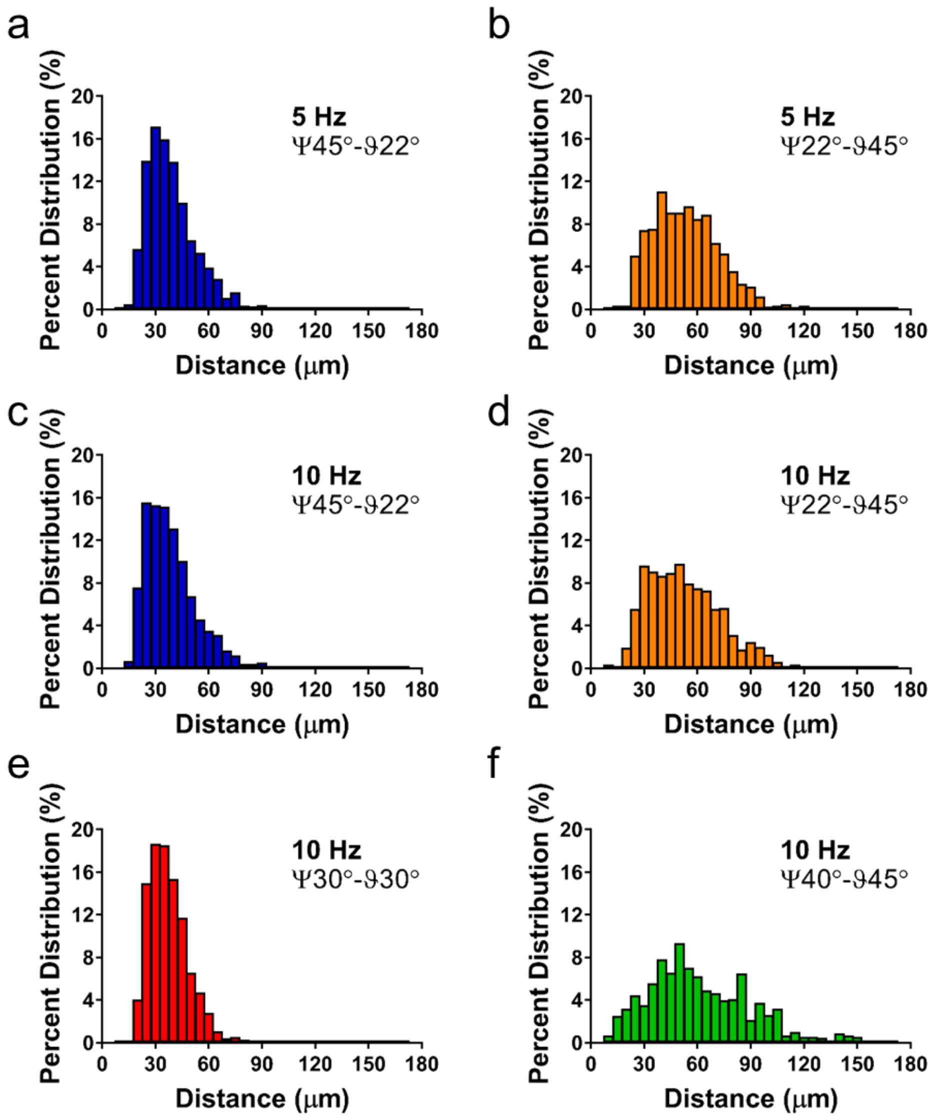

Figure S8. Nearest neighbor distributions for microrobot swarms. (a-f) Percent distributions of nearest neighbor distance in microrobot swarms formed at different tilt and precession angles and actuated at 5 Hz and 10 Hz.

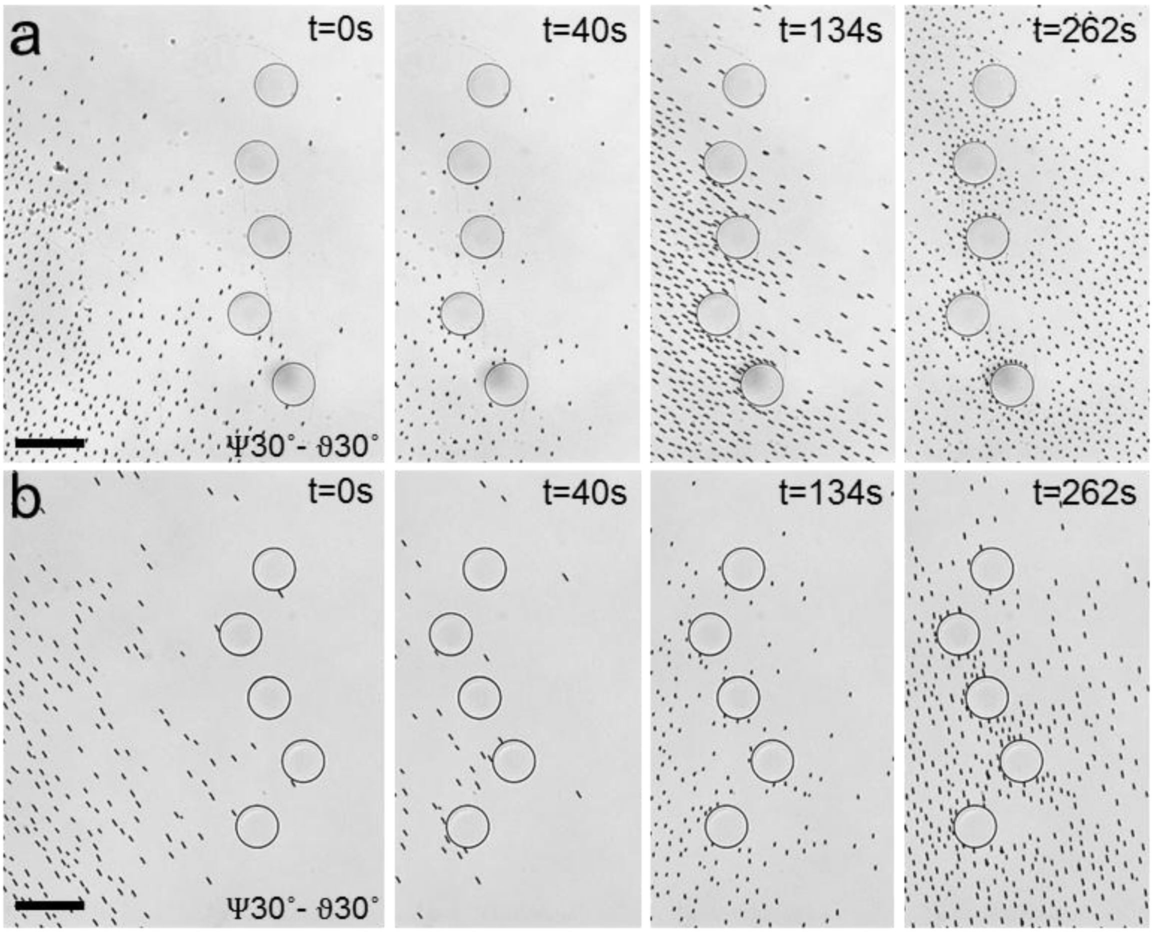

Figure S9. Locomotion of microrobotic swarms through confining obstacles with varying gap distances. Microrobotic swarms traversing an array of obstacles with a vertical distance of **(a)** 90 µm and **(b)** 60 µm. Scale bar is 200 µm.

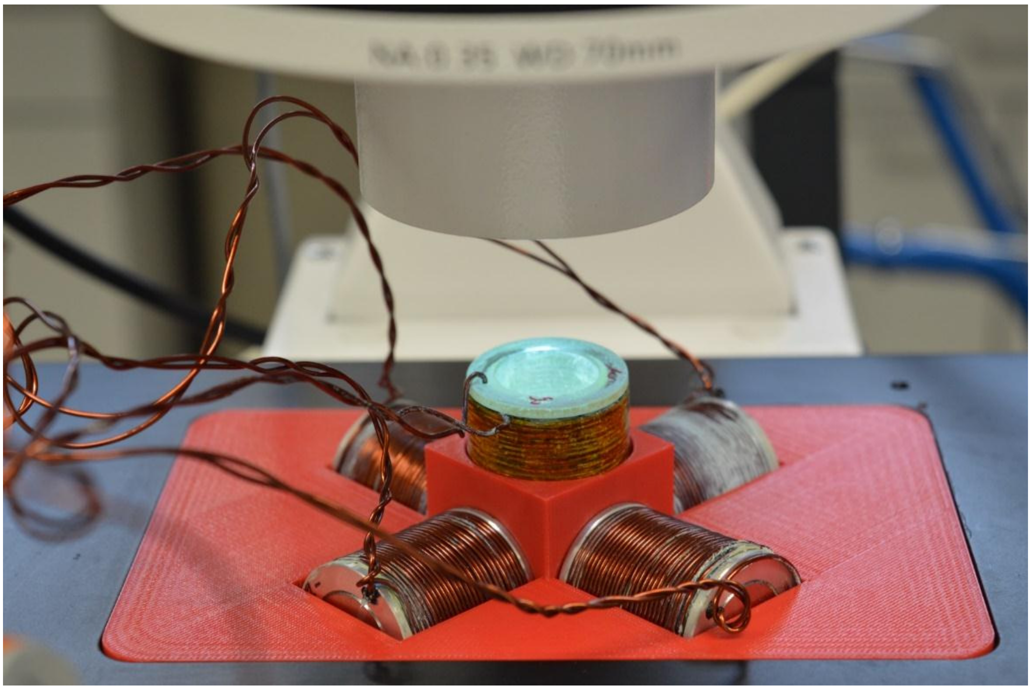

Figure S10. Photo of the experimental setup composed of custom-designed five electromagnetic coils mounted on an inverted optical microscope.

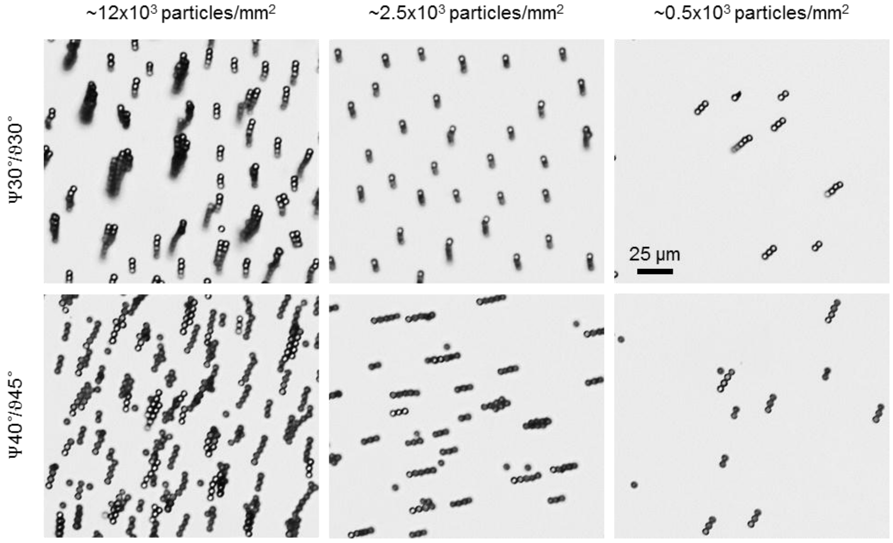

Figure S11. Effect of initial particle concentration on dynamic self-assembly and organization of microrobots. High particle concentration (~12x10^3 particles/mm^2) led to formation of thick columnar structures along with chains, whereas low particle concentrations (~0.5x10^3 particles/mm^2) resulted in sparsely distributed chains in the workspace. Direction of the inter-robot interactions (repulsive/attractive) were independent of initial particle concentration.

Supplementary Movies

Movie S1. Precession (ψ), tilt (ϑ), and orientation angles (α) of the applied magnetic fields. w indicates the precession axis about which a time-varying magnetic field (B(t)) precesses.

Movie S2. Reversible assembly and propulsion of microrobot swarms. Dispersion, assembly, propulsion and disassembly sequences were performed consequently in one continuous experiment (~10 minutes). Video is edited for brevity. p: pointing direction, t: translation direction, w: precession axis direction, ω: angular velocity.

Movie S3. Guided propulsion of a microrobot consisting of self-assembled chain of magnetic microparticles. p: pointing direction, t: translation direction, w: precession axis direction, ω: angular velocity.

Movie S4. Simulation of microrobot propulsion. A planar solid surface is located at z=0.

Movie S5. Collective order in dynamically self-assembled microrobotic swarms

Movie S6. Locomotion of microrobotic swarms through confining obstacles

Movie S7. Directed cargo transport using microrobotic swarms. In transport of large cargoes on the surface, a mixture of fluorescent poly(methyl methacrylate) (PMMA) particles with sizes varying between 1 µm to 20 µm were used. Particle speeds up to 2 µm/s were observed. In bulk transport of small tracers, polystyrene particles of around 1 µm were used. Speeds up to 8 µm/s were observed for 1 µm particles.
